# Supplementary material for: Chromothripsis during telomere crisis is independent of NHEJ, and consistent with a replicative origin
Source: Genome Res. 2019 May;29(5):737–49. doi: 10.1101/gr.240705.118 (PMC6499312; doi:10.1101/gr.240705.118)
Supplement: Supplemental Material [file supp_gr.240705.118_Supplemental_file_1.zip › contigs/annotated_contigs/DB51/contig.2.DB51_length_268_mean_cov_6.15671641791.docx]

**DB51_length_268_mean_cov_6.15671641791**

```
GACAGCAATTTCAGTGAGTTTTAGAGCAGACCTTTCCCTGGTTGCGTCTCAATGTGGGAATGCAGCCTGGATGCCACGTTGATCGCAGC
 >chr18:68956044-68956238 + E=9e-103
CTTGTGGTATCTGGAGCCCACGGTCAAGTGATGCAGCACTGGGAGGGATTCCTTTCCTACGGAAACAGTGAGCCAGTCAGTGTGTATTG

TTAGAAGCTGCTAAT|A|ACACAAGGTCAGGCTCCTGGCACCAGAGGGAGAGCCAGGCAGGCATAAACTGCACACGGGTCAGACAAGAG
                >chr18:68906560-68906635 + E=7e-34
CGA
```
